# Concentration patterns of antibacterial factors and immunoglobulin A antibody in foremilk fractions of healthy cows

**DOI:** 10.1111/asj.13372

**Published:** 2020-04-13

**Authors:** Nana Kitano, Naoki Isobe, Jun Noda, Toshihiko Takahashi

**Affiliations:** ^1^ Graduate School of Dairy Science Rakuno Gakuen University Ebetsu Hokkaido Japan; ^2^ Graduate School of Integrated Science for Life Hiroshima University Higashi‐Hiroshima Japan; ^3^ Graduate School of Veterinary Science Rakuno Gakuen University Ebetsu Hokkaido Japan

**Keywords:** antibacterial factor, foremilk, immunoglobulin A antibody, lactating cow

## Abstract

Antibacterial factors act as innate immune components, which respond as soon as bacteria enter a living organism. To prevent and treat mastitis in cattle, understanding the concentrations of these substances inside the udder is important; however, they remain to be studied. In this investigation, the concentration of lingual antimicrobial peptide (LAP), S100 protein (S100A7), lactoferrin (LF), and immunoglobulin antibody were measured in the different fractions of foremilk. Lactating Holstein cows were examined, and 10 foremilk fractions were obtained from sequential samples up to 150 ml. The LAP concentrations in milk samples increased until 25 ml. The LF concentrations increased up to the 10 ml fraction, then stabilized at low level after the 50 ml fraction. For S100A7, some fractions had significantly higher (*p* < .05) concentrations than the 5 or 10 ml fractions. The IgA antibody concentration increased up to the 5 ml fraction, then after 50 ml fraction showed relatively low concentrations. This investigation determined the concentration patterns of LAP, LF, S100A7, and IgA antibody secreted in milk inside the udders of healthy lactating cows as baseline data. These distinct concentration patterns might indicate various protective responses.

## 1 INTRODUCTION

Antibacterial factors (AFs), such as antimicrobial peptides and proteins, belong to the innate immune system, which functions as soon as bacteria enter a living organism in a healthy condition. Innate immune reactions are much faster than those of the acquired immune system; a common immune system utilizing immunoglobulins (antibodies), which require a few days to respond after bacterial penetration. Antibodies react with bacteria in a specific manner, whereas AFs can attack a broad spectrum of bacteria and viruses using lytic functions (Schonwetter, Stolzenberg, & Zasloff, 1995). AFs are intrinsically present in animals, and they have a low potential for the emergence of resistant strains (Levy et al., 2015). AFs are synthesized in different organs in ruminant animals (Mach & Pahud, 1971). Furthermore, the AFs can target a broad spectrum of microbes such as bacteria, fungi, and viruses. More specifically, defensin, S100A7, and lactoferrin proteins are synthesized in the lactating gland, and knowing the concentrations of such components is important.

The lingual antimicrobial peptide (LAP) was reported to be expressed at the gene level at the lactating gland and is increased to express under the mastitis condition (Swanson et al., 2004). Furthermore, it has been reported that LAP is localized in the mammary gland, galactophore, and epithelial cells (Isobe, Hosoda, & Yoshimura, 2009a). S100A7 is a Ca‐binding protein, and it possesses antimicrobial function (Tetens et al., 2010). Also, S100A7 has been reported to be localized in mammary epithelial cells, the mucosal layer inside the teat, and the epithelium of the teat skin (Zhang, Lai, Yoshimura, & Isobe, 2014). The experimental intramammary infusion of lipopolysaccharide into the mammary gland in goats increased S100A7 abundance in the mammary papilla cutaneous epithelium and significantly increased its concentration in milk (Zhang et al., 2014). From this, S100A7 was expected to demonstrate antimicrobial function at the bacterial entry point of the mammary gland and papilla.

Another response under the mastitis condition is an expression of lactoferrin (LF) in mammary gland epithelial cells, where it may sequester iron to minimize the multiplication of bacteria resulting in bacteriostasis. Additionally, for immune globulins (antibodies) with acquired immunity, when udder infection occurs with pathogen invasion, immunoglobulin levels increase in the lacteal in response to the sensitized antibody as an essential indicator (Boothby, Jasper, & Thomas, 1987; Nashar, Stokes, & Cripps, 1991; Newby & Bourne, 1977; Yokomizo & Norcross, 1978). The increased number of immunoglobulin‐producing cells suggested the induction of local immune responses in the mammary gland and papilla (Nickerson, Baker, & Trinidad, 1989). For the group of antibodies, the report stated that an increased level of IgA‐positive cells in mastitis affected the mammary part, and it suggested that the response of IgA antibody secretion on bacteria attributed to mastitis within the mucous membrane of the mammary papilla in an infected udder (Nickerson & Heald, 1982). Furthermore, an IgA plays a vital role in the mucous membrane, where it is mainly secreted into the intestinal tract as a secretory IgA to react with pathogens. In the mammary gland, secretory IgA‐producing plasma cells are distributed, and secretory IgA is known to be secreted in milk. This kind of IgA is called as natural IgA that binds antigen nonspecifically (de Klerk et al., 2015). However, the distribution pattern of AF and IgA in the udder was not considered. Therefore, it is necessary to understand the presence and secretion pattern of AFs in the lactating gland as an initial step. This investigation tried to reveal the AF and immunoglobulin concentration pattern inside the udder lacteal by measuring different fractions of foremilk samples focused on determining baseline data of AFs and antibody marker components with healthy cows.

## 2 MATERIAL AND METHOD

### 2.1 Milk sampling

The examined Holstein cows belonged to Rakuno Gakuen University Field Education Research Center with a free‐stall barn as the rearing environment. All the examined cows showed no clinical sign of mastitis at least 1 month before the experiment.

The different lactation stages of cows were examined with single udders for each individual cows. Table 1. indicates a detail description of the examined individual cows for one prime, two middle, and four late lactation periods. The parities were one to three (average 1.6 ± 0.8) calves. The series of foremilk samples were obtained after the predipping process of removing moisture with a towel. All the foremilk samples were obtained between 16:00 and 18:00, and prior milking was between 5:00 and 6:00; thus, ca. 12 (±1) h. of the nonmilking period was allocated. The foremilk samples were collected with following fractions in series: 5 ml, 10 ml, 15 ml, 20 ml, 25 ml, 50 ml, 75 ml, 100 ml, 125 ml, and 150 ml. The fractions intervals were, 5 ml = 0–5 ml, 10 ml = 5–10 ml, 15 ml = 10–15 ml, 20 ml = 15–20 ml, 25 ml = 20–25 ml, 50 ml = 45–50 ml, 75 ml = 70–75 ml, 100 ml = 95–100 ml, 125 ml = 120–125 ml, and 150 ml = 145–150 ml.

**TABLE 1:** The background information of examined cows: days after calving, lactation stage, and parity

*n *＝ 7	Days after calving	Lactation stage	Parity
No 1	85	Prime	1
No 2	148	Middle	1
No 3	162	Middle	3
No 4	218	Late	2
No 5	219	Late	2
No 6	241	Late	1
No 7	242	Late	1

### 2.2 AF and immunoglobulin in the foremilk sample

The following components in samples were analyzed by ELISA techniques: for the AF, LAP. LF, and S100A7, and for IgA. The measurement methods for LAP, LF, and S100A7 were in accordance with Isobe, Morimoto, Nakamura, Yamasaki, and Yoshimura (2009b), Isobe, Shibata, Kubota, and Yoshimura (2013), and Zhang et al. (2014), respectively. The IgA antibody concentrations were determined using the method of Matsukawa, Ueno, and Sugino (2018).

### 2.3 Statistical analysis

The values are indicated as the mean and standard deviation. Statistical analyses were performed using a commercial software package (IBM SPSS Statistics, v.21, IBM Co, Somers, NY, USA). Nonparametric multiple comparisons, Friedman's test was employed to determine statistically significant differences. A value of *p* < .05 was considered to be statistically significant.

## 3 RESULT

### 3.1 AF concentrations in the foremilk samples

**FIGURE 1:**
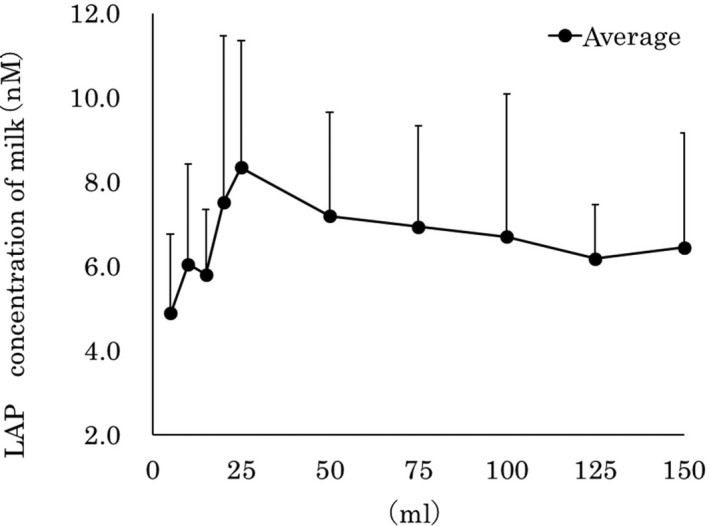
LAP concentrations in different fractions of foremilk samples

Figure 1 shows the LAP concentrations in the different fractions of foremilk samples. The average concentrations of LAP (n*M*) were, 5 ml: 4.9 ± 1.1, 10 ml: 6.1 ± 2.4, 15 ml: 5.8 ± 1.5, 20 ml: 7.5 ± 4.0, 25 ml: 8.3 ± 3.0, 50 ml: 7.2 ± 2.5, 75 ml: 6.9 ± 2.4, 100 ml: 6.7 ± 3.4, 125 ml: 6.2 ± 1.3, and 150 ml: 6.5 ± 2.7. The LAP concentrations in the 5 ml to 25 ml fractions of foremilk samples showed an increasing trend; thereafter, the concentrations decreased gradually.

**FIGURE 2:**
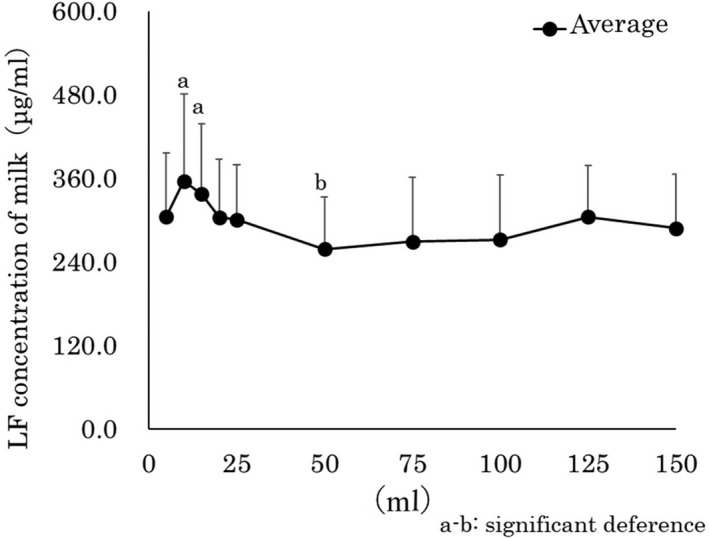
LF concentrations in different fractions of foremilk samples

Figure 2 shows LF concentrations in the different fractions of foremilk samples. The average concentrations of LF (μg/mL) were, 5 ml: 305.0 ± 90.9, 10 ml: 356.1 ± 125.1, 15 ml: 337.1 ± 101.2, 20 ml: 303.8 ± 83.6,25 ml: 300.4 ± 79.1,50 ml: 258.5 ± 74.9, 75 ml: 269.3 ± 91.7, 100 ml: 271.8 ± 93.4, 125 ml: 304.8 ± 73.7, and 150 ml: 288.0 ± 78.0. The LF concentration in the foremilk samples showed an increasing trend up to the 10 ml fraction, a decreasing trend up to the 50 ml fraction, then the rest of the fractions had relatively low concentrations.

**FIGURE 3:**
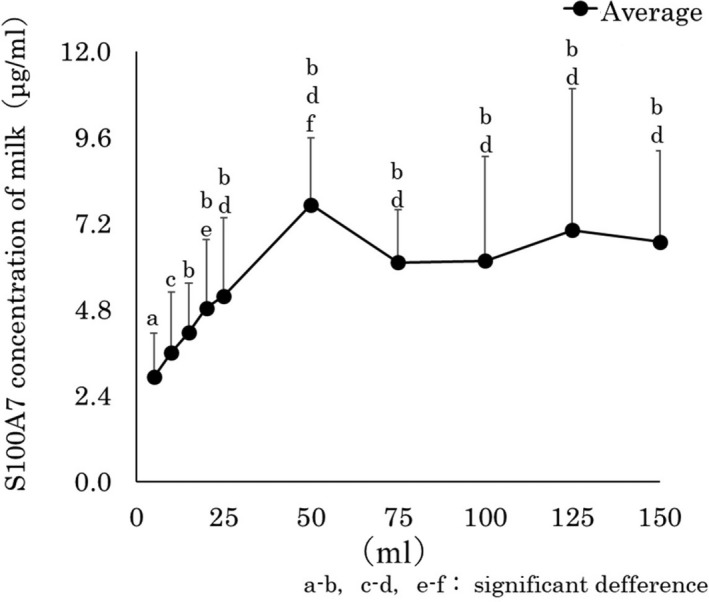
S100A7 concentrations in different fractions of foremilk samples

Figure 3 shows the S100A7 concentrations in the different fractions of foremilk samples. The average concentrations of S100A7 (μg/mL) were, 5 ml: 2.9 ± 1.2, 10 ml: 3.6 ± 1.7, 15 ml: 4.2 ± 1.4, 20 ml: 4.8 ± 1.9, 25 ml: 5.2 ± 2.2, 50 ml: 7.7 ± 1.9, 75 ml: 6.1 ± 1.5, 100 ml: 6.2 ± 2.9, 125 ml: 7.0 ± 3.9, and 150 ml: 6.7 ± 2.5. The comparison of S100A7 concentrations, the 50 ml, 125 ml, and 150 ml fractions had significantly higher values (*p* < .05) than the 5 ml fraction. Also, for the 50 ml and 150 ml fractions had significantly higher values (*p* < .05) than the 10 ml fraction. Lastly, for the 50 ml fraction had significantly higher values (*p* < .05) than the 50 ml fraction.

The S100A7 concentration in the foremilk samples showed an increasing trend up to the 50 ml fraction, then the fractions up to 150 ml had relatively high concentrations.

**FIGURE 4:**
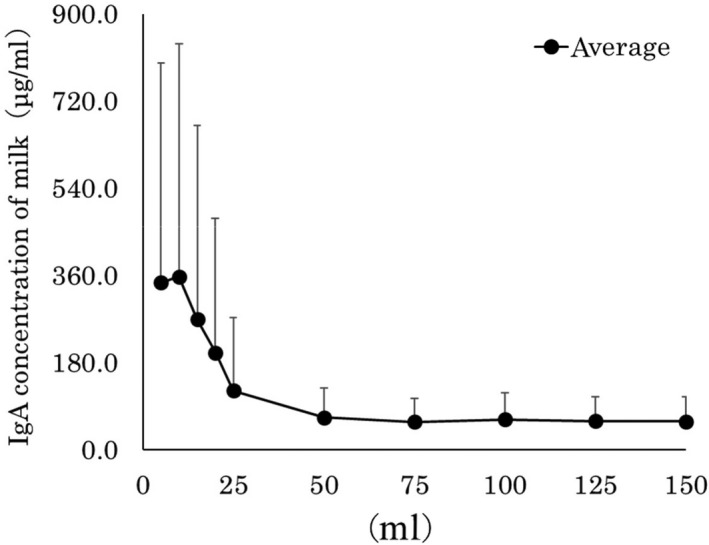
IgA antibody concentrations in different fractions of foremilk samples

Figure 4 shows the IgA concentrations in the different fractions of foremilk samples. The average concentrations of IgA antibodies (μg/mL) were, 5 ml: 345.1 ± 453.7, 10 ml: 357.4 ± 481.3, 15 ml: 269.2 ± 400.8, 20 ml: 200.7 ± 277.6, 25 ml 122.0 ± 151.1, 50 ml: 66.8 ± 60.9, 75 ml: 57.7 ± 48.7, 100 ml: 62.6 ± 55.4, 125 ml: 59.5 ± 50.8, and 150 ml: 58.9 ± 51.3.

The comparison of IgA concentrations, the 75 ml, 100 ml, and 150 ml fractions had significantly lower values (*p* < .05) than the 5 ml fraction. The IgA concentrations in the examined foremilk were relatively high up to 10 ml, and then the fractions after 50 ml had relatively low concentrations.

## 4 DISCUSSION

This investigation examined AF (LAP, LF, and S100A7) and IgA concentration patterns in milk samples inside the udder of cows from the foremilk fraction. The result of quantitative measurements indicated that the concentration of LAP increased up to the 25 ml fraction, then after the 50 ml fraction, it stabilized at relatively low concentrations. The results of the LAP concentration pattern suggested that LAP is secreted in the deeper part of the udders, such as the mammary gland and epithelial cells of the galactophore, which agreed with a previous report (Huang et al., 2012). In a prior investigation with the injection of lipopolysaccharide into the udder, the concentration of LAP increased in milk samples; in the same investigation, injection of PBS did not cause an increase in the concentration of LAP (Isobe, Morimoto, et al., 2009). From this study, the examined cows were healthy animals, and the determined LAP concentrations were stable in the foremilk samples. A variation in LAP concentrations occurs most probably with the intrusion of mastitis, which was caused by bacteria entering the udder, as described in Swanson et al. (2004). From the results and literature information, it was reasonable to observe there was no variation in LAP concentrations in the foremilk samples.

The LF concentration showed an increasing trend, and the results were more stable after the 50 ml fraction. According to previous reports, LF concentrations depend highly on the age of cows, stage of lactation, and affected mastitis condition (Hagiwara, Kawai, Anri, & Nagahata, 2003). Therefore, the foremilk sample up to 10 ml indicated an increasing trend for LF, which may have been caused by a defense reaction against bacterial intrusion from the mouth of the mammary papilla as an early sign of mastitis.

The comparison of S100A7 concentrations, above the 50 ml, fractions had significantly higher values (*p* < .05) than the 5 ml, 10 ml, and 15 ml fractions. S100A7 was reported to be expressed in the lower udder section (Zhang et al., 2014), and it is expected to be found at a high concentration in earlier fraction then gradually decrease in the later fraction of foremilk samples; however, the result was the opposite. This result can be explained by greater bacterial invasion near the mouth of the mammary papilla, which increased the consumption/utilization of S100A7. Possibly, there are other mechanisms to reduce the S100A7 level; however, an investigation to understand such mechanism is beyond the scope of this study.

The IgA antibody concentrations in the foremilk samples indicated the 75 ml, 100 ml, and 150 ml fractions had significantly lower values (*p* < .5) than the 5 ml and 10 ml fractions. IgA plays an important role in mucosal immunity; it is also abundant in milk and distributed in the udder. IgA behaves as in the intestinal tract, most probably by B‐cell mobilization from the blood vessel into organs first, then secreted IgA, which is transported to milk. From the result of this investigation, the high titer of IgA was observed in the first (5 ml) fraction of the foremilk, because a large number of B cells exist near the mammary papilla. However, somatic cells also contain lymph corpuscles, and it is also possible that IgA may be secreted from B cells within the milk.

From the above results, the patterns of AF concentration were different. From this, the secreted AFs (LAP, LF, S100A7) and immunoglobulin (IgA antibody) are responsible for diverse protective responses in the udder of healthy milking cows.

This study only focused on determining baseline data of AFs and antibody marker components with healthy cows. Because we had no access to cows with mastitis in this investigation, further comparisons to investigate similar measurements in such cows will be necessary. From the result obtained relatively stable values of AF and were observed after 25 ml to 50 ml fractions of foremilk samples. These stable fractions may be reasonable to compare with similar measurements with mastitis‐affected cows. The further acquisition of similar data sets for mastitis‐affected cows will bring more in‐depth knowledge of the antibody marker components concentrations to be utilized for the preventative approach and early‐stage treatment.
